# Genetic Variations in *MDM2* Gene Contribute to Renal Cell Carcinoma Susceptibility: A Genotype–Phenotype Correlation Study

**DOI:** 10.3390/cancers17020177

**Published:** 2025-01-08

**Authors:** Shu-Yu Chang, Wen-Shin Chang, Hou-Yu Shih, Chao-Hsiang Chang, Hsi-Chin Wu, Chia-Wen Tsai, Yun-Chi Wang, Jian Gu, Da-Tian Bau

**Affiliations:** 1 Graduate Institute of Biomedical Sciences, China Medical University, Taichung 404333, Taiwan; 2 Terry Fox Cancer Research Laboratory, Department of Medical Research, China Medical University Hospital, Taichung 404327, Taiwan; 3 Department of Nephrology, Chang-Hua Hospital, Ministry of Health and Welfare, Changhua 51341, Taiwan; 4 Department of Epidemiology, The University of Texas MD Anderson Cancer Center, Houston, TX 77030, USA; 5 School of Medicine, China Medical University, Taichung 40402, Taiwan; 6 Department of Bioinformatics and Medical Engineering, Asia University, Taichung 413305, Taiwan

**Keywords:** genotype, MDM2, phenotype, renal cell carcinoma, single nucleotide polymorphism (SNP), Taiwan

## Abstract

Background: This study aimed to investigate the polymorphic genotypes of *MDM2* rs937282, rs937283, rs2279744, and rs769412, as well as the combined effects of *MDM2* genotypes and environmental factors on RCC susceptibility. Methods: A total of 135 RCC patients and 590 controls were recruited for *MDM2* genotyping using the polymerase chain reaction-restriction fragment length polymorphism (PCR-RFLP) method. Quantitative PCR was performed to assess MDM2 mRNA levels among 30 healthy individuals and 22 RCC patients. Results: *MDM2* rs2279744, but not other polymorphisms, was significantly associated with an increased RCC risk (*p* = 0.0133). The *MDM2* rs2279744 G allele was identified as a risk factor for RCC (odds ratio [OR] = 1.49, 95% confidence interval [CI] = 1.14–1.96, *p* = 0.0047). Among smokers (*p* = 0.0070), alcohol drinkers (*p* = 0.0233), individuals with hypertension (*p* = 0.0041), diabetes (*p* = 0.0225), and those with a family history of cancer (*p* = 0.0020), the *MDM2* rs2279744 GT and GG genotypes exhibited increased RCC risks. However, this risk effect was not observed in non-smokers, non-drinkers, or individuals without hypertension, diabetes, or a family cancer history (all *p* > 0.05). Moreover, MDM2 mRNA levels were significantly higher in RCC patients compared to controls and varied among the rs2279744 genotypes, with GG genotype exhibiting the highest expression levels among both RCC patients and controls. Conclusions: This study highlights the association between *MDM2* rs2279744 genotypes and RCC risk, suggesting that genotype-associated MDM2 mRNA levels could contribute to early RCC detection. Further studies are warranted to elucidate the detailed mechanisms underlying the role of *MDM2* in RCC development.

## Simple Summary

The objective of the current study was to investigate the contribution of *mouse double minute 2* (*MDM2*) genotypes and phenotypes to the risk of renal cell carcinoma (RCC). Our findings indicate that the *MDM2* rs2279744 G allele serves as a risk marker against RCC. In addition, it can interact with environmental and clinical risk factors, such as smoking, alcohol drinking, hypertension, and diabetes, to influence RCC risk. Furthermore, MDM2 mRNA levels were significantly higher in RCC patients compared to controls and varied among the *MDM2* rs2279744 genotypes, with the GG genotype exhibiting the highest expression levels in both RCC patients and controls. These genotype-associated MDM2 mRNA levels may serve as a practical marker for early RCC detection, alongside *MDM2* rs2279744 genotypes. This combined approach could offer potential benefits for personalized and precise early prediction and detection of RCC, which currently lacks effective early detection methodologies.

## 1. Introduction

Renal cell carcinoma (RCC), the most prevalent solid tumor of the kidney, represents approximately 3% of all malignancies worldwide [1,2,3]. RCC is the most common renal malignancy and encompasses up to 21 subtypes. These subtypes are distinguishable by their unique histopathological features, genetic underpinnings, clinical progression, and therapeutic responses [4,5,6]. Numerous lifestyle factors, including sedentary behavior, obesity, insufficient vegetable consumption, tobacco use, and alcohol intake, have been identified as contributors to RCC risk [7]. RCC often progresses asymptomatically, particularly in advanced stages, leading to delayed diagnosis [8,9]. Furthermore, up to 30% of patients who undergo radical nephrectomy experience significant complications and face early recurrence [10]. Current prognostic assessments for RCC heavily rely on histological evaluations, which are time-consuming and often provide limited guidance for selecting optimal therapeutic strategies [11]. Consequently, there is an urgent need for robust molecular genomic markers to enable the early detection of RCC. Although recent studies have highlighted several hereditary factors associated with RCC susceptibility [12,13,14,15,16], the complex interplay between genetic predispositions and environmental or lifestyle factors remains poorly understood and warrants further investigation.

The *murine double minute 2 (MDM2)* is a gene situated on human chromosome 12q14.3-q15, spanning 34 kb and encoding a 491-amino-acid protein. The promoter region of *MDM2* harbors several single nucleotide polymorphisms (SNPs), including rs937282, rs939283, and rs2279744, which have been implicated in modulating MDM2 expression and influencing cancer risk. Among these, rs2279744 is located within the second promoter-enhancer region of *MDM2* and involves a T-to-G substitution at position 309 of intron 1. This SNP creates a binding site for the transcription factor Sp1, resulting in increased MDM2 expression and the subsequent suppression of the p53 pathway in cells exposed to DNA-damaging agents [17,18]. Rs2279744 is the most extensively studied *MDM2* SNP regarding its role in human diseases. Beyond rs2279744, several other SNPs, such as rs937282, rs939283, rs117039649, rs3730485, and rs769412, have also been investigated for their associations with cancer susceptibility. Both rs937282 and rs939283 are located in the *MDM2* promoter region, whereas rs117039649 and rs3730485 exhibit high linkage disequilibrium with rs2279744. Rs769412, in contrast, is an exonic SNP in exon 11 of the *MDM2* gene.

Over recent years, the association between *MDM2* SNPs and RCC risk has been explored in only three studies, involving Japanese, Chinese, and Caucasian populations [19,20,21]. Haitel et al. identified the *MDM2* rs2279744 GG genotype as significantly associated with an elevated RCC risk [19]. Huang et al. confirmed similar findings, reporting an association between rs2279744 genotypes and RCC susceptibility [20]. Conversely, de Martino and colleagues found no significant relationship between the *MDM2* rs2279744 and RCC risk, nor with tumor stage, grade, or histological subtype [21]. Importantly, no other SNPs in *MDM2* have been evaluated in relation to RCC. From a proteomic perspective, early evidence from Haitel and colleagues demonstrated that the MDM2 protein is overexpressed in RCC tissues and serves as a marker of poor prognosis and reduced survival [22]. Later, a Japanese research group corroborated these findings, showing that individuals with the rs2279744 GG genotype exhibited the highest levels of MDM2 protein expression compared to those with GT or TT genotypes [19]. However, no studies to date have investigated the influence of *MDM2* genotypes, including rs2279744, on MDM2 mRNA expression levels in RCC patients.

**Figure 1:**
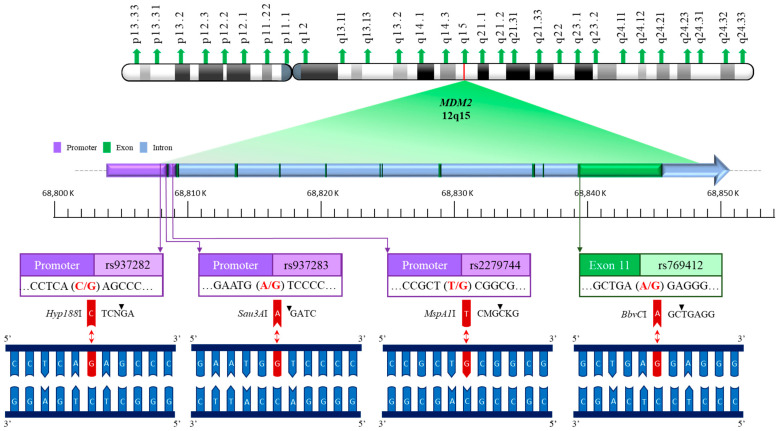
Physical map of *MDM2* rs937282, rs937283, rs2279744, and rs769412 polymorphic sites on part of human chromosome 12 (12q15). The restriction enzymes and cutting points are also shown.

In this study, we comprehensively analyzed the genotypes of *MDM2* promoter SNPs rs937282, rs937283, and rs2279744, along with the exonic SNP rs769412, in a cohort of Taiwanese patients with RCC. The chromosomal locations of these SNPs are illustrated in Figure 1. For the first time, we investigated the potential interactions between *MDM2* genotypes and environmental or clinical risk factors associated with RCC susceptibility. Additionally, we measured MDM2 mRNA expression levels in RCC patients and healthy controls to elucidate the genotype–phenotype relationship.

## 2. Materials and Methods

### 2.1. Selected Subjects

This case–control study was conducted at China Medical University Hospital and included 135 patients diagnosed with RCC and 590 cancer-free controls, matched for age and gender. None of the participants were genetically related. RCC diagnoses, as well as tumor grades and subtypes, were confirmed through histopathological evaluation by an experienced group of surgeons and pathologists led by Drs. Wu and Chang.

The cancer-free control subjects were recruited from the Health Examination Center of China Medical University Hospital. Initially, each RCC patient was frequency-matched to 4–5 controls of the same gender and within ±2 years of age. However, individuals with incomplete demographic data regarding smoking, alcohol consumption, hypertension, diabetes, or family history of cancer were excluded. Additional exclusion criteria for controls included any symptoms suggestive of RCC, such as hematuria. After applying all these criteria, a total of 590 eligible controls were retained for analysis.

Each participant provided written informed consent, and 3–5 mL of venous blood was collected for DNA and RNA extraction and subsequential genotyping and RT-PCR. The study protocols have been approved by the Institutional Review Board of China Medical University Hospital (approval number: DMR98-IRB-209). The overall participation rate exceeded 85%. Key demographic and clinical characteristics of the RCC cases and controls are summarized and compared in Table 1.

### 2.2. DNA Extraction and MDM2 Genotyping Methodology

Genomic DNA was extracted from peripheral blood leukocytes of each participant utilizing a QIAamp Blood Mini Kit according to the guidance from the manufacturer (Qiagen, Valencia, CA, USA). Then, the extracted DNA was stored at −80 °C for long-term preservation. All procedures adhered to standardized protocols routinely employed in our laboratory [15,23,24,25,26].

Genotyping of *MDM2* rs937282, rs937283, rs2279744, and rs769412 was performed using the polymerase chain reaction-restriction fragment length polymorphism (PCR-RFLP) methodology. The PCR conditions included an initial denaturation step at 94 °C for 5 min, followed by 35 cycles of 94 °C for 30 s, 59 °C for 30 s, and 72 °C for 30 s, with a final extension at 72 °C for 10 min. Upon completion of the reaction, the PCR amplicons were promptly collected and verified by DNA electrophoresis to confirm the expected product sizes. The sequences of the forward and reverse primers used for each SNP are summarized in Table 2.

Once PCR verification was completed, the amplified products were subjected to enzymatic digestion. Specific restriction enzymes—*Hyp188*I for rs937282, *Sau3A*I for rs937283, *MspA1*I for rs2279744, and *BbvC*I for rs769412—were utilized for cleavage. Digestion was performed overnight to ensure complete enzymatic processing. The resulting DNA fragments were then analyzed by electrophoresis on 3.0% agarose gel, run at 100 V for 30 min. Table 2 provides an overview of the primers, restriction enzymes, and the sizes of DNA fragments before and after digestion. The accuracy of these PCR-RFLP assays was confirmed in our pilot testing of 10 samples using Sanger sequencing. The genotypes at these four SNP sites were 100% concordant between the PCR-RFLP and sequencing assays.

### 2.3. MDM2 RNA Level Measurement by Quantitative Real-Time PCR

A total of 52 blood samples, comprising 22 from RCC cases and 30 from non-cancer healthy subjects, were subject to RNA extraction utilizing RNeasy Kits (Qiagen, Valencia, CA, USA) following the manufacturer’s protocol. Complementary DNA (cDNA) was synthesized from 1 µg of total RNA of each sample using the RT2 First Strand Kit (Qiagen). Real-time quantitative PCR (qPCR) was performed to evaluate *MDM2* RNA expression utilizing an FTC-3000 real-time PCR system (Funglyn Biotech Inc., Toronto, ON, Canada). *Glyceraldehyde 3-phosphate dehydrogenase* (*GAPDH*) was used as the internal standard for normalization. The primer sequences were designed as follows: forward 5′-GAAATCCCATCACCATC-TTCCAGG-3′ and reverse 5′-GAGCCCCAGCCTTCTCCATG-3′ for the *GAPDH* gene; forward 5′-TGTAAGTGAACATTCAGGTG-3′ and reverse 5′-TTCCAATAGTCAGCTAAGGA-3′ for the *MDM2* gene. The qPCR reactions were performed in a total volume of 20 µL, containing IQ™ SYBR Green Supermix Master Mix (Bio-Rad, Hercules, CA, USA), 0.5 µmol/L of each primer, and 9 ng of cDNA. Each sample was analyzed independently three times to ensure accuracy. The relative MDM2 expression levels were quantified by the typical 2^−ΔΔCt^ method, normalized to *GAPDH* expression, and shown as the average of three independent experiments. This methodology was the same as our previous publication [27].

### 2.4. Statistical Analysis

The final statistical analysis included data from 590 cancer-free controls and 135 RCC cases, all with complete genotypic and demographic information. To ensure the representativeness of the control group for the Taiwanese population and to exclude potential genotyping errors, the genotype frequencies of each *MDM2* polymorphism in the control group were evaluated for Hardy–Weinberg equilibrium using a goodness-of-fit test. Pearson’s Chi-square test was applied to compare the distribution of *MDM2* genotypes between the RCC case and control subjects, as well as across stratified subgroups. Differences in continuous variables, such as age and MDM2 RNA expression levels, were analyzed using the unpaired Student’s *t*-test. Logistic regression analysis was performed to assess the association between *MDM2* genotypes and RCC risk, with results expressed as odds ratios (ORs) and 95% confidence intervals (CIs). Any *p*-value output below 0.05 was considered statistically significant.

## 3. Results

### 3.1. Comparison of Characteristics Between RCC Patients and Healthy Controls

The epidemiological and clinical characteristics of the 135 RCC patients and 590 cancer-free controls are summarized and compared in Table 1. Due to matching by age and gender, no significant differences were observed between the groups for these variables (*p* = 0.4769 and *p* = 0.9567, respectively). Additionally, the two groups showed no statistically significant differences in terms of smoking, alcohol consumption, or diabetes prevalence (*p* = 0.7519, *p* = 0.5491, and *p* = 0.1323, respectively).

As expected, the RCC group exhibited significantly higher proportions of individuals with hypertension (66.9% vs. 50.2%) and a family history of cancer (9.3% vs. 3.1%) compared to the controls (*p* = 0.0004 and *p* = 0.0005, respectively). Histopathological analysis revealed that 77.1% of RCC cases were of the clear cell subtype. Regarding tumor grade, 53.4% were classified as low-grade, while 46.6% fell into the middle- and high-grade categories (Table 1).

### 3.2. Association Between MDM2 Genotypes and RCC Risk in Taiwan

The genotypic frequencies of *MDM2* rs937282, rs937283, rs2279744, and rs769412 were analyzed in 135 RCC patients and 590 age- and gender-matched healthy controls (Table 3). Hardy–Weinberg equilibrium testing showed that the genotype distributions of all four SNPs in the control group were consistent with expected frequencies (*p*-values = 0.2197, 0.6448, 0.9035, and 0.3040, respectively).

A significance in the distributions of *MDM2* rs2279744 CC, CG, and GG genotypes was identified between the RCC and control groups by the trend analysis (*p* for trend = 0.0133) (Table 3, middle part). Noticeably, individuals carrying the heterozygous GT or homozygous GG genotypes of *MDM2* rs2279744 exhibited an elevated risk of RCC, with ORs of 1.35 (95% CI = 0.79–2.30, *p* = 0.3346) and 2.13 (95% CI = 1.22–3.71, *p* = 0.0098), respectively. Under the dominant model, the combined GT + GG genotypes of *MDM2* rs2279744 demonstrated a borderline association with RCC risk (OR = 1.63, 95% CI = 0.98–2.69, *p* = 0.0732, Table 3, middle panel). In contrast, no significance was observed between RCC risk and any of the genotypes of *MDM2* rs937282, rs937283, or rs769412 (Table 3).

Further allelic frequency analysis of these four *MDM2* genotypes is presented in Table 4. The results confirmed that the variant allele G of *MDM2* rs2279744 was present in 61.9% of RCC patients, significantly higher than in the control group (52.1%) (OR = 1.49, 95% CI = 1.14–1.95, *p* = 0.0047), corroborating the findings from Table 3. Conversely, no significant association was found between RCC susceptibility and the other three SNPs (*MDM2* rs937282, rs937283, and rs769412) (Table 4).

### 3.3. Stratified Analysis of MDM2 rs2279744 Genotypes According to Epidemiological and Clinical Risk Factors

We then conducted stratified analyses based on epidemiological and clinical risk factors, including smoking, alcohol consumption, hypertension, diabetes, and family history of cancer, according to individuals’ *MDM2* rs2279744 genotypes.

**Figure 2:**
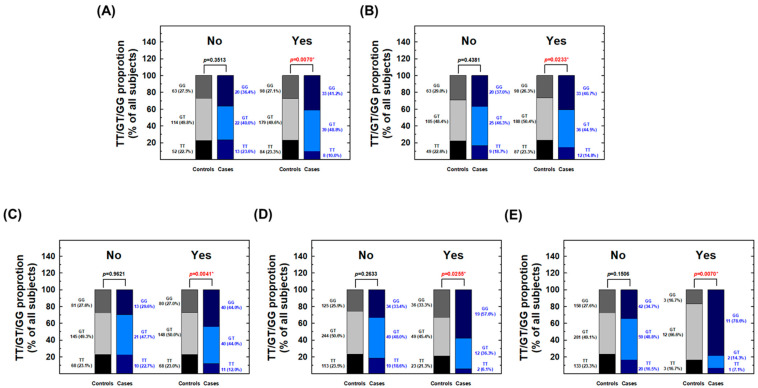
Genotype distributions of *MDM2* rs2279744 in cases and controls as stratified by the status of (**A**) smoking, (**B**) alcohol drinking, (**C**) hypertension, (**D**) diabetes, and (**E**) family history of cancer. Statistically significant *p*-values between case and control groups are shown in red and asterisk.

First, a significant difference in the genotypic distribution of *MDM2* rs2279744 was observed between RCC cases and controls among smokers (*p* = 0.0070) but not among non-smokers (*p* = 0.3513, Figure 2A). Among smokers, the frequency of the rs2279744 GG genotype was markedly higher in the RCC group compared to the control group (41.2% vs. 27.1%, Figure 2A), suggesting the effect of the GG genotype on RCC risk is only evident in smokers but not in non-smokers.

Second, the genotypic distribution differed significantly between RCC cases and controls among alcohol drinkers but not among non-drinkers (Figure 2B). Specifically, alcohol drinkers in the RCC group had a higher frequency of the GG genotype compared to controls (40.7% vs. 26.3%, Figure 2B). This suggests that the risk of the GG genotype was pronounced in alcohol drinkers but absent in non-drinkers.

Third, stratification by hypertension status revealed a significant association between the rs2279744 GG genotype and elevated RCC risk among hypertensive individuals (*p* = 0.0041). However, no such association was observed in non-hypertensive individuals (*p* = 0.9621, Figure 2C).

Fourth, an analysis stratified by diabetes status showed that the risk of the rs2279744 GG genotype was evident in diabetic individuals (*p* = 0.0225) but not in non-diabetic subjects (*p* = 0.2633, Figure 2D).

Finally, an analysis stratified by family history of cancer revealed a differential distribution of *MDM2* rs2279744 genotypes only among those with a family history of cancer (*p* = 0.0020, Figure 2E), suggesting that the risk of the GG genotype of *MDM2* rs2279744 is particularly relevant in this subgroup.

### 3.4. The Expression Levels of MDM2 mRNA According to Individual Genotypes

**Figure 3:**
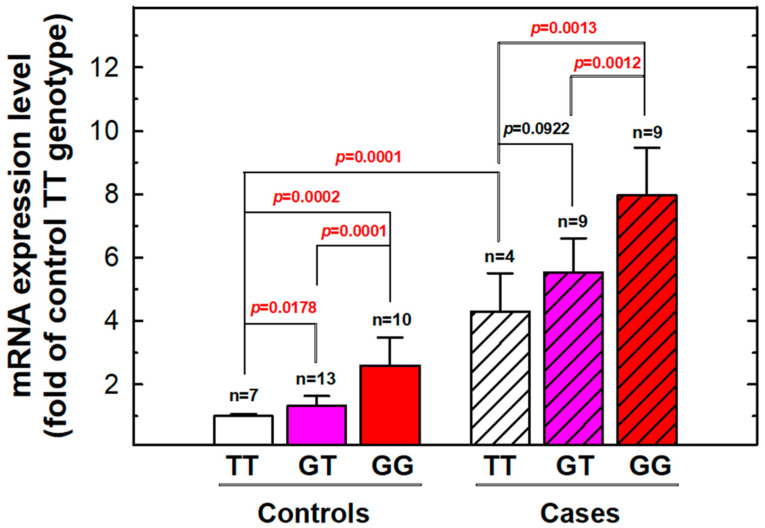
Serum MDM2 mRNA levels among 22 RCC patients and 30 healthy control subjects. The serum MDM2 mRNA levels were measured by the quantitative PCR methodology. The relative levels of serum mRNA MDM2 were based on folds of those carrying the *MDM2* rs2279744 TT genotype for the controls. Statistically significant *p*-values between compared groups are shown in red.

The blood levels of *MDM2* in 22 RCC patients and 30 healthy controls were measured and are illustrated in Figure 3. The relative MDM2 mRNA expression was significantly elevated in RCC patients compared to healthy controls (*p* = 0.0001, Figure 3). Based on their *MDM2* rs2279744 genotypes, these individuals were categorized into three subgroups: TT genotype, GT genotype, and GG genotype. In both the case and control groups, a significant trend was observed: MDM2 mRNA levels increased with the number of variant G alleles. The homozygous variant genotype group (GG) exhibited the highest MDM2 mRNA levels, the heterozygous variant genotype group (GT) showed intermediate levels, and the wild-type genotype group (TT) displayed the lowest MDM2 mRNA expression (Figure 3).

## 4. Discussion

Clinically, early-stage RCC often presents with minimal or non-specific symptoms, making its early detection particularly challenging. The identification of reliable genomic biomarkers could significantly enhance the precision of RCC risk assessment and outcome prediction. Recent studies have highlighted that *MDM2* genotypes may influence serum MDM2 levels, potentially contributing to cancer susceptibility. These findings have been reported across multiple cancer types, supporting the importance of *MDM2* as a potential biomarker [28,29,30]. Polymorphic variations at *MDM2 rs2279744* have been associated with either increased or decreased cancer risk in various contexts, including retinoblastoma [31], oral cancer [32], esophageal cancer [33,34], lung cancer [35,36,37,38,39,40,41], breast cancer [42,43], gastric cancer [44,45,46], hepatocellular carcinoma [47,48,49], colorectal cancer [50,51,52], endometrial cancer [53,54,55,56], cervical cancer [57], prostate cancer [58], melanoma [59], and childhood acute lymphoblastic leukemia [60]. Similarly, the *MDM2* rs937282 polymorphism has been linked to lung cancer [38] and breast cancer [61], while *MDM2* rs937283 has been implicated in retinoblastoma [62], oral cancer [32], thyroid cancer [63], breast cancer [61,64,65], gastric cancer [66], and liver cancer [65]. In addition, *MDM2* rs769412 has been associated with the risks of lung cancer [67] and breast cancer [68], while no significant correlations have been observed in oral cancer [69], lung cancer [41], or retinoblastoma [62]. Taken together, the collective evidence supports a potential link between *MDM2* genotypes and RCC susceptibility, warranting further investigation into their role as predictive biomarkers in this malignancy.

In this study, we conducted a comprehensive investigation of the *MDM2* SNPs rs937282, rs937283, rs2279744, and rs769412 among 135 RCC patients and 590 healthy controls from the Taiwanese population. Taiwan’s geographical isolation and genetic homogeneity, combined with distinctive lifestyle factors such as widespread traditional herbal medicine use, have contributed to a higher incidence of RCC compared to other regions.

Our findings revealed a significantly increased RCC risk associated with the heterozygous GT and homozygous GG genotypes of *MDM2* rs2279744 (Table 3 and Table 4). Conversely, no significant associations were observed between RCC susceptibility and the genotypes of *MDM2* rs937282, rs937283, or rs769412 (Table 3 and Table 4). These results support the hypothesis that *MDM2* rs2279744 genotypes may serve as a susceptibility factor for RCC. The findings align with previous reports by Huang et al. and Hirata et al. [19,20] but contradict the negative results from de Martino et al., which were based on a Caucasian population [21]. The discrepancy may be attributed to genetic, lifestyle, and environmental differences between East Asian populations and Caucasian populations. It is worth pointing out that the frequency of the variant G allele of rs2279744 varies dramatically among different races and ethnicities, with East Asians showing by far the highest prevalence: 10.8% in Africans, 36.3% in Europeans, and 53.3% in East Asians (Japanese and Koreans) (https://www.ncbi.nlm.nih.gov/snp/rs2279744, accessed on 3 January 2025). The G allele frequency in our controls (52.1%) aligns with that of East Asians.

This current study also presents two novel findings. First, *MDM2* rs2279744 genotypes were not only predictors of RCC susceptibility but also showed significant interactions with risk factors such as smoking, alcohol consumption, hypertension, diabetes, and a family history of cancer (Figure 2). Second, the G allele of *MDM2* rs2279744 was associated with elevated MDM2 mRNA expression levels in both RCC patients and healthy individuals (Figure 3). The integration of *MDM2* genotypes with personal behavioral and clinical factors offers potential for personalized RCC risk prediction and early detection. Additionally, the correlation between *MDM2* rs2279744 and mRNA expression levels highlights the potential utility of combining DNA and RNA analyses for precise and non-invasive diagnostic strategies. Future research should validate the contribution of *MDM2* genotypes, particularly rs2279744, to RCC risk in larger and more diverse populations globally. Such studies could solidify the role of *MDM2* SNPs in RCC risk assessment and pave the way for their integration into clinical practice.

A detailed stratification analysis of RCC patients and controls based on epidemiological and clinical characteristics revealed that *MDM2* rs2279744 genotypes significantly influenced RCC susceptibility among smokers (Figure 2A), alcohol consumers (Figure 2B), individuals with hypertension (Figure 2C), diabetes (Figure 2D), and those with a family history of cancer (Figure 2E). However, no significant association was observed for non-smokers, non-drinkers, or individuals without hypertension, diabetes, or a family history of cancer (Figure 2). A previous study by Huang and colleagues also examined the association between *MDM2* rs2279744 genotypes and RCC risk, reporting that the GT and GG genotypes were linked to RCC susceptibility after adjusting for confounding factors such as age, gender, body mass index, smoking, alcohol consumption, tea and coffee drinking, and hypertension [21]. Their study cohort, which had similar genotypic frequency distributions to ours, consisted of a smaller sample size (127 cases and 254 controls) compared to the present investigation. Despite the differences in cohort size, the similarity in findings underscores the potential role of *MDM2* rs2279744 in RCC risk modulation within this population.

The genotype–phenotype correlation was analyzed by measuring serum MDM2 levels in 22 RCC patients and 30 healthy controls. The results revealed significant differences in MDM2 serum levels among individuals with different *MDM2* rs2279744 genotypes in both controls and cancer patients: serum MDM2 mRNA levels increased progressively with the number of variant G alleles. The homozygous variant genotype group (GG) exhibited the highest levels of MDM2 mRNA, followed by the heterozygous genotype group (GT) with intermediate levels and the wild-type genotype group (TT) with the lowest expression (Figure 3). Additionally, MDM2 mRNA levels were significantly elevated in cancer patients compared to controls. These findings align with a previous study on gastric cancer [70]. These findings suggest that the *MDM2* rs2279744 genotype could serve as a convenient biomarker for assessing RCC risk and progression. Furthermore, the associated RNA expression levels, and, potentially, protein levels in future studies, may help address the challenges of early-stage RCC detection and prediction. This dual approach of genotype and phenotype assessment could significantly enhance early diagnostic capabilities for RCC in clinical practice.

The *MDM2* gene encodes a protein containing several conserved functional domains, including an N-terminal p53-binding domain, a bipartite nuclear localization sequence, a nuclear export sequence, an acidic domain, and a C-terminal RING finger domain, critical for its E3 ubiquitin ligase activity [71,72]. MDM2 is a well-known negative regulator of p53 that directly binds to and inhibits p53. Overexpression of MDM2 has been reported in several cancers, including RCC [73,74]. Overexpression of MDM2 is also a worse prognosis marker for RCC [74]. The regulation of MDM2 is intricate and context-dependent at multiple levels, including genetic (gene amplification), transcriptional/post-transcriptional (e.g., SNP309, promoter CpG methylation, non-coding RNA, alternative splicing, and mRNA stability), and translation/post-translational regulation (e.g., phosphorylation, ubiquitination, and SUMOylation) [75]. SNP rs2279744 is one of the regulation mechanisms of MDM2 expression. At the molecular level, the T-to-G substitution at *MDM2* rs2279744 has been reported to create an Sp1 transcription factor binding site, thereby enhancing MDM2 expression. Bond et al. [76] conducted pioneering work on the function of SNP in vitro. They confirmed the binding of Sp1 to the SNP region, and found MDM2 mRNA and protein levels were significantly higher in cell lines with homozygous (G/G) (on average 4-fold) and heterozygous genotypes at rs2279744 (T/G) (on average 1.9-fold) than cell lines with the wild-type genotype (T/T). The increase in MDM2 levels attenuates the p53 tumor suppressor pathway, thereby accelerating tumor initiation and progression [76,77]. This suggests that the GG genotype at MDM2 rs2279744 may elevate MDM2 expression, disrupt p53 function, and contribute to RCC development and progression. In addition to inhibiting p53, MDM2 exerts other oncogenic effects. For instance, siRNA-mediated downregulation of MDM2 has been shown to reduce the expression of HIF1α and HIF2α in VHL-defective RCC [78]. Mutations in VHL and the subsequent activation of HIF1α and HIF2α are key drivers of RCC development. Targeting MDM2 as a therapeutic strategy for cancers has been extensively studied, and several Phase 3 clinical trials are currently underway [79]. Although our study does not directly measure MDM2 protein levels, the observed increase in MDM2 mRNA associated with the GG genotype (Figure 3) provides indirect support for this hypothesis. Hirata et al. [19] showed that the variant G allele of rs2279744 led to increased MDM2 expression in RCC tumors; furthermore, the homozygous G/G genotype is an independent predictor of worse cancer-specific survival. Our study is the first study to link the rs2279744 genotype with RCC risk in the Taiwanese population and the first to show increased serum MDM2 levels in RCC patients as well as a significant genotype-mRNA expression in the context of RCC initiation. The SNP and serum mRNA level may facilitate identifying high-risk individuals and early detection of RCC. We could not assess the prognostic value of rs2279744 and MDM2 in our patient cohort, due to limited sample size, heterogeneous treatment, and short follow-up time. The precise molecular mechanisms underlying the interactions between *MDM2* and other key regulatory proteins, such as p53 and p21, in the context of RCC remain unclear. Future research is necessary to identify other factors regulating MDM2 regulation in RCC, assess the prognostic value of rs2270744 in RCC patients, and perform high-throughput sequencing in tumor DNA/RNA to discover mutations and gene expression alterations related to RCC progression and survival.

## 5. Conclusions

In summary, this pilot study identifies a significant correlation between the *MDM2* rs2279744 genotypes and RCC susceptibility in the Taiwanese population. Notably, this is the first study to explore the interaction between *MDM2* genotypes and behavioral or clinical factors in relation to RCC risk. Our findings indicate that the *MDM2* rs2279744 SNP is significantly associated with RCC susceptibility, particularly among individuals who smoke, consume alcohol, or have comorbidities such as hypertension, diabetes, or a family history of cancer. These results underscore the potential utility of *MDM2* genotyping in facilitating early RCC detection, especially in high-risk individuals.

## Figures and Tables

**Table 1:** Distributions of the frequencies of selected characteristics among the renal cell carcinoma patients and healthy controls.

Characteristic	Cases (n = 135)		Controls (n = 590)		*p*-Value
	n	%	n	%	
Age, mean ± SD	59.0 ± 9.6		58.2 ± 9.9		0.4769
≤60 Years	70	51.7%	310	52.5%	0.9606
>60 Years	65	48.3%	280	47.5%	
Sex					
Male	86	64.4%	380	64.4%	0.9567
Female	49	35.6%	210	35.6%	
Smoking status					
Smoker	55	40.7%	229	38.8%	0.7519
Non-smoker	80	59.3%	361	61.2%	
Alcohol drinking status					
Drinker	54	41.5%	217	36.8%	0.5491
Non-drinker	81	58.5%	373	63.2%	
Hypertension					
Yes	91	66.9%	296	50.2%	0.0004 *
No	44	33.1%	294	49.8%	
Diabetes					
Yes	33	22.0%	108	18.3%	0.1323
No	102	78.0%	482	81.7%	
Family history cancer					
Yes	14	9.3%	18	3.1%	0.0005 *
No	121	90.7%	572	96.9%	
Histological type					
Clear-cell	105	77.1%			
Non-clear-cell	30	22.9%			
Histological grade					
Low	72	53.4%			
Middle and high	63	46.6%			

Statistically significant *p*-values based on chi-square test with Yates’ correction are shown in bold with a star.

**Table 2:** The summary of primer sequences, restriction enzyme, and DNA fragment sizes for *mouse double minute 2 (MDM2)* rs937282, rs937283, rs2279744, and rs769412 polymorphic sites.

Polymorphisms	Primer Sequences	RE	Polymorphic Genotype	DNA Fragment Size (bp)
rs937282	F: 5′-GGTAACAGCGACACGGAGAT-3′ R: 5′-CGCATCCGGGCATTTGTGC-3′	*Hyp188*I	C G	307 182 + 125
rs937283	F: 5′-CGGATTAGTGCGTACGAGCG-3′ R: 5′-TCAGAGCCCAGACCCAAAAG-3′	*Sau3A*I	G A	202 154 + 48
rs2279744	F: 5′-TTCGCAGCCTTTGTGCGGTT-3′ R: 5′-GAACGTGTCTGAACTTGACC-3′	*MspA1*I	T G	368 223 + 145
rs769412	F: 5′-GGTTACAGAAACTGACTGTG-3′ R: 5′-CACATCTTCTTGGCTGCTAT-3′	*BbvC*I	A G	371 196 + 175

F and R indicate forward and reverse primers, respectively; RE: restriction enzyme.

**Table 3:** Associations between *MDM2* genotypes and RCC risk in Taiwan.

Polymorphism	Genotype	Cases	Controls	*p*-Value	OR (95% CI)
rs937282	CC	74 (54.8%)	320 (54.2%)		1.00 (Ref)
	CG	47 (34.8%)	221 (37.5%)	0.7610	0.92 (0.61–1.38)
	GG	14 (10.4%)	49 (8.3%)	0.6376	1.24 (0.65–2.36)
*P*_trend_				0.6861	
*P*_HWE_				0.2197	
	CG + GG	61 (45.2%)	270 (45.8%)	0.9795	0.98 (0.67–1.42)
rs937283	AA	73 (54.1%)	331 (56.1%)		1.00 (Ref)
	AG	50 (37.0%)	219 (37.1%)	0.9454	1.04 (0.70–1.54)
	GG	12 (8.9%)	40 (6.8%)	0.4942	1.36 (0.68–2.72)
*P*_trend_				0.6829	
*P*_HWE_				0.6448	
	AG + GG	62 (45.9%)	259 (43.9%)	0.7400	1.09 (0.75–1.58)
rs2279744	TT	21 (15.5%)	136 (23.0%)		1.00 (Ref)
	GT	61 (45.2%)	293 (49.7%)	0.3346	1.35 (0.79–2.30)
	GG	53 (39.3%)	161 (27.3%)	0.0098 *	2.13 (1.22–3.71)
*P*_trend_				0.0133 *	
*P_HWE_*				0.9035	
	GT + GG	114 (84.5%)	454 (77.0%)	0.0732	1.63 (0.98–2.69)
rs769412	AA	130 (96.3%)	561 (95.1%)		1.00 (Ref)
	AG	5 (3.7%)	28 (4.7%)	0.7650	0.77 (0.29–2.03)
	GG	0 (0.0%)	1 (0.2%)	1.0000	--
*P*_trend_				0.7758	
*P_HWE_*				0.3040	
	AG + GG	5 (3.7%)	29 (4.9%)	0.7076	0.74 (0.28–1.96)

RCC: renal cell carcinoma; OR: odds ratio; CI: confidence interval; *p*-values were calculated by Chi-square with Yates’ correction (n ≥ 5) or Fisher’s exact test (n < 5); HWE: *P*_HWE_: *p*-value for Hardy–Weinberg Equilibrium; *P*_trend_: *p*-value for trend analysis; statistically significant *p*-values are shown in bold with a star.

**Table 4:** Associations of *MDM2* alleles with RCC risk in Taiwan.

Allelic Type	Cases	Controls	*p*-Value	OR (95% CI)
rs937282				
C	195 (72.2%)	861 (73.0%)		1.00 (Ref)
G	75 (27.8%)	319 (27.0%)	0.8634	1.04 (0.77–1.40)
rs937283				
A	196 (72.6%)	881 (74.7%)		1.00 (Ref)
G	74 (27.4%)	299 (25.3%)	0.5325	1.11 (0.83–1.50)
rs2279744				
T	103 (38.1%)	565 (47.9%)		1.00 (Ref)
G	167 (61.9%)	615 (52.1%)	0.0047 *	1.49 (1.14–1.95)
rs769412				
A	265 (98.1%)	1150 (97.5%)		1.00 (Ref)
G	5 (1.9%)	30 (2.5%)	0.6548	0.72 (0.28–1.88)

RCC: renal cell carcinoma; OR: odds ratio; CI: confidence interval; *p*-value was calculated by Chi-square with Yates’ correction; statistically significant *p*-values are shown in bold with a star.

## Data Availability

The data that support the findings of this study are available on request from the corresponding author. The data are not publicly available due to privacy or ethical restrictions.
